# New light on changes in the number and function of blood platelets stimulated by cocoa and its products

**DOI:** 10.3389/fphar.2024.1366076

**Published:** 2024-03-12

**Authors:** Beata Olas

**Affiliations:** Department of General Biochemistry, Faculty of Biology and Environmental Protection, University of Lodz, Lodz, Poland

**Keywords:** blood platelets, cocoa, flavanols, flavonoids, phenolic compounds

## Abstract

Hyperactivation of blood platelets, one of the causes of heart attack, and other cardiovascular diseases (CVDs), is influenced by various dietary components, including phenolic compounds from vegetables, fruits, teas, wines, cocoa and its products, including chocolate. The present paper sheds new light on the effect of cocoa and its products, especially dark chocolate, on the number and function of blood platelets, and the anti-platelet activity of their constituent phenolic compounds. A review was performed of papers identified in various electronic databases, including PubMed, Science Direct, Scopus, Web of Knowledge, and Google Scholar, with the aim of determining whether their anti-platelet activity may serve as part of a sweet strategy in countering CVDs. Various studies demonstrate that cocoa consumption, especially in the form of dark chocolate, with a high flavanol concentration, has anti-platelet activity and may play a significant role in cardioprotection; they also note that cocoa consumption may be a good strategy in diminishing cardiovascular risk, including hyperactivation of blood platelets.

## Introduction

Cardiovascular diseases (CVDs), disorders related to the heart and circulatory system, include various disease units (Zhang et al., 2019; Desai et al., 2023). Among them, coronary artery disease and cerebrovascular artery disease are two of the most common causes of death globally (Zhang et al., 2019; Desai et al., 2023). For many of these conditions, especially stroke and ischemic heart disease, the etiology is based around atherosclerosis and blood platelet activation.

Blood platelets are small, discoid-shaped circulatory cells. Their cell membrane contains various receptors (including glycoprotein GPIIb/IIIa, also known as integrin α_IIb_β_3_), that regulate the interaction between blood platelets, leukocytes, and intracellular matrix. Upon activation (inducted by various agonists, including thrombin), blood platelets change their shape and release more adhesion receptors, exposing them on their surface (Sang et al., 2021). Although blood platelets are known to play an important role in developing atherothrombosis, its occurrence is also associated with hypertension, smoking, abnormal cholesterol levels, physical inactivity, diabetes, obesity, and a family history of CVD (Teissedre et al., 2018; Bittner, 2020). However, it has been found that blood platelet activation can also be modulated by certain dietary components, including phenolic compounds from herbs, vegetables, fruits, teas and wines, as well as cocoa and its products, including chocolate (Kerimi and Williamson, 2015; Sperkowska et al., 2021).

A number of *in vitro*, *in vivo* and epidemiological studies indicate that diets rich in flavonoids, a subgroup of phenolic compounds, may protect against CVDs by inhibiting blood platelet function (Mozaffarian, 2016). A number of epidemiological studies (Almoosawi et al., 2012; Arranz et al., 2013; Ferri et al., 2015; Vlachojannis et al., 2016; Dong et al., 2017; Lee et al., 2017; Loffredo et al., 2017; Barrios et al., 2018; Garcia et al., 2018; Rees et al., 2018; Zięba et al., 2019; Latif and Majeed, 2020; Tyc et al., 2021; Seecheran et al., 2022), highlight a relationship between the consumption of flavanol-rich cocoa products and a lower incidence of CVDs, including ischemic heart disease and stroke. These studies included consumption of various cocoa products, including dark chocolate, milk chocolate, cocoa powder, and drinks with theobromine. Most importantly, consumption of cocoa and its products was often associated with positive modulation of blood platelet-mediated hemostasis and CVDs (Seecheran et al., 2022). Although a number of review papers indicate that cocoa and its products have cardioprotective potential, these present little information about their anti-platelet action (Fernandez-Murga et al., 2011; Andjuar et al., 2012; Senturk and Gunay, 2015; Loffredo et al., 2017; Gammone et al., 2018; Santos and Macedo, 2018; Zięba et al., 2019; Ghaffari and Roshanravan, 2020). While other studies have found the phenolic compounds from cocoa and its products to have anti-platelet activity, little is known of the mechanisms behind it (Almoosawi et al., 2012; Dong et al., 2017; Lee et al., 2017; Loffredo et al., 2017; Barrios et al., 2018; Latif and Majeed, 2020; Otreba et al., 2021; Seecheran et al., 2022).

The aim of the present study was to review the most recent literature concerning the possible mechanisms behind the anti-platelet activity of the phenolic compounds in cocoa and its products, especially dark chocolate. It sheds new light on the effect of cocoa and its products on blood platelet number and function. It presents a review of 70 review papers and 254 research papers published over the last 20 years. The papers were drawn from various electronic databases, including PubMed, Science Direct, Scopus, Web of Knowledge, and Google Scholar, with extra papers identified by manually reviewing the references. The search was restricted to English language publications (reviews and articles). The following terms were used: *cocoa, cocoa product, blood platelet, platelet, flavonoid,* and *cardiovascular disease*. The last search was run on 8 February 2024.

## Cocoa and chocolate–chemical content

Although cocoa and chocolate are two different terms, their main component is the cocoa bean (*Theobroma cocoa*). The term *cocoa* refers to the natural products, i.e., the non-fat component of cocoa liquor, the pure extract of cocoa beans. Although chocolate is a processed food, in some countries such as Spain, it is also taken as a beverage (Fernandez-Murga et al., 2011; Andjuar et al., 2012; Senturk and Gunay, 2015; Tyc et al., 2021). Cocoa contains about 380 known chemical compounds (Fernandez-Murga et al., 2011; Andjuar et al., 2012).

Cocoa and its products are widely consumed throughout the world. Of these products, chocolate is the most popular, and due to its unique texture and teste, it is usually consumed for pleasure. Chocolate is a confectionery product made from cocoa beans, cocoa lipids, and sugar; however, it often contains other ingredients, including nuts, milk, coffee, alcohol and fruits, typically raisins. The type of chocolate depends on the content of cocoa beans, added sugar and other ingredients. Dark chocolate contains the most cocoa beans (50%–85% of total weight), followed by dessert chocolate (30%–70%) and milk chocolate (20%–30%). Some regional differences exist: commercially-produced dark chocolate has about 15% cocoa in the USA and 35%–50% in Europe (Senturk and Gunay, 2015). White chocolate does not contain cocoa beans, but only cocoa butter, fat, milk and sugar.

Cocoa and its products are rich sources of bioactive compounds. For example, cocoa contains about 30%–50% lipids, with this value consisting of approximately 30% stearic acid (saturated fatty acid) and 25% palmitic acid (saturated fatty acid), as well as 30% oleic acid: an unsaturated fatty acid which may play an important role in the prevention and treatment of CVDs through different mechanisms, including inhibition of blood platelet activation (Olas, 2020). Moreover, stearic acid exerts a neutral cholesterolemic response in humans (Fernandez-Murga et al., 2011).

Another important group of ingredients in chocolate, representing up to 10% of the dry weight of the bean, comprises phenolic compounds. However, their precise content depends on the manufacturing process (Andjuar et al., 2012; Senturk and Gunay, 2015; Sperkowska et al., 2021) and its place of origin, for example, the concentration of catechin is 16.52 mg/g in Costa Rican cocoa, and 2.66 mg/g in cocoa grown in Jamaica (Andjuar et al., 2012). The phenolic compound content is also reduced by several production processes, including fermentation, storage, drying and roasting (Loffredo et al., 2017).

Phenolic compounds can be classified according to their chemical structure, biological properties and source of origin. For example, according to Vermerris and Nicholson (Vermerris and Nicholson, 2006), these compounds can be classified into three groups: 1) simple phenols and phenolic acids, 2) flavonoids (including flavanones, flavonols, flavanols, anthocyanins, flavones, and isoflavones), and 3) other polyphenols. Flavonoids are made up of three rings A, B, and C; they usually occur in nature in a bound form, most often as glycosides. An important subclass of flavonoids are the flavanols, which differ from the other flavonoids by the presence of an OH group attached to the C_3_ carbon in the heterocyclic C ring, and the lack of double bonds and carbonyl groups.

In addition, chocolate is also recommended as a valuable source of other compounds, including vitamins, minerals (especially magnesium, and phosphorus), trace elements and low concentrations of theobromine (Senturk and Gunay, 2015; Davinelli et al., 2018; Montagna et al., 2019; Febrianto et al., 2021). The chemical composition of dark chocolate is presented in Table 1. It important to note that cocoa also contains small amounts of fiber and plant sterols, which may contribute to lower serum lipids (Fernandez-Murga et al., 2011).

**TABLE 1:** Chemical content of dark chocolate (Sperkowska et al., 2021).

Chemical compounds	Chocolate
Water (g)	0.6
Protein (g)	6.7
Lipid (g)	34.3
Stearic acid (mg/g cocoa fat)	0.9
Palmitic acid (mg/g cocoa fat)	203.8
Oleic acid (mg/g cocoa fat)	304
Linoleic acid (mg/g cocoa fat)	27.8
Carbohydrate (g)	56.6
Sugar (g)	38.3
Total fiber (g)	1.7
Theobromine (g/kg)	10
Sodium (µg)	4,000
Potassium (µg)	581,000
Iron (µg)	21,000
Calcium (µg)	42,000
Phosphorum (µg)	244,000
Thiamin (µg)	40
Riboflavin (µg)	10
Total phenolic compounds (ng/gallic acid equivalent (GAE)/L)	11.7–14.8
Catechin (mg/kg)	107–500
Epicatechin (mg/kg)	32,7–125
Quercetin (mg/kg)	250
Ferulic acid (mg/kg)	240
Resveratrol (mg/kg)	0.4

## Intake of phenolic compounds and their bioavailability

The intake of phenolic compounds, including flavonoids, varies greatly from population to population, and the daily intake of flavonoids is believed to range from 20 to 650 mg per day in terms of aglycan content. In addition, it has been suggested that the mean flavonoid consumption is 189.7 mg per day, of which as much as 83.5% are flavanols (catechins), 7.6% flavanones, 6.8% flavonols and 1.6% anthocyanins (Williamson and Holst, 2008; Loffredo et al., 2017; Azad et al., 2021). For example, Williamson and Holst (Williamson and Holst, 2008) note that >500 mg of phenolic compounds daily can be obtained from “five-a-day,” i.e., consuming five portions of fruit or vegetables each day. Consumption of cocoa could easily increase this by 500–1,000 mg. Recently, Crowe-White et al. (Crowe-White et al., 2022) have described that consumption of 400–600 mg/day flavan-3-ols can reduce risk associated with CVDs.

**FIGURE 1:**
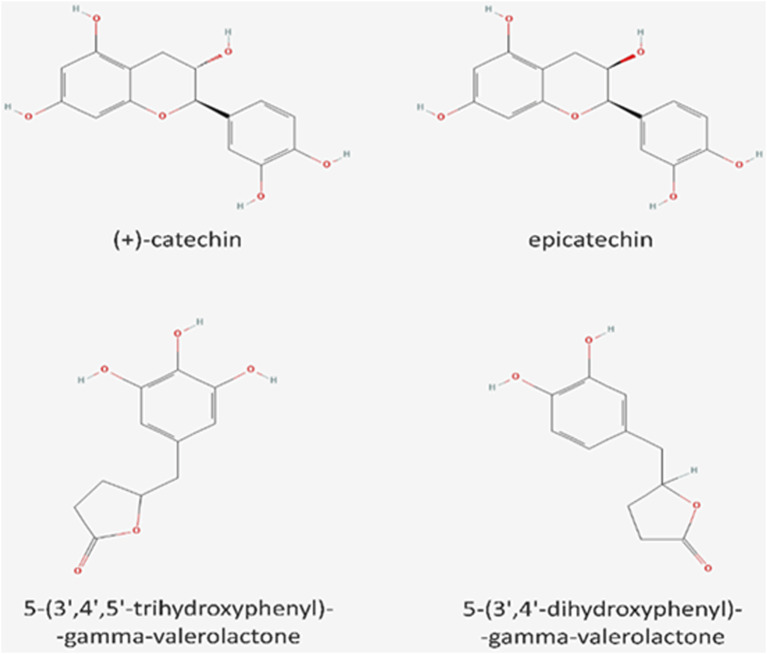
The chemical structure of the main flavanols in cocoa: catechin and epicatechin and their metabolites (5-(3′,4′,5′-trihydroxyphenyl)-gamma-valerolactone and 5-(3′,4′-dihydroxyphenyl)-gamma-valerolactone).

Flavonoids, including flavanols (catechins), are generally considered to be the main phenolic compounds in cocoa and chocolate. Catechin, epicatechin, and their analogs (gallocatechins) are abundant in cocoa beans (1.4 g/kg). Flavanols are also found in many other plant foods, like grape seeds, grapes, apples, apricots, green tea, black tea, and red wines (Murkovic, 2016; Prasain et al., 2018) (Table 2). According to the USA Department database (USDA), 100 g of blueberries yields 3 mg of flavanols while 100 g of apple yields 9 mg (Williamson and Holst, 2008). After oral ingestion, they are processed by the gut microflora, resulting in the generation of a large variety of metabolites. Flavanols reach their peak concentration within two to 3 hours after intake; the final concentration is dose dependent, and the products are detectable in the plasma even after 8 hours (Dugo et al., 2018). For example, Li et al. (Li et al., 2000) detected two catechin metabolites in the plasma and urine of human volunteers: 5-(3′,4′,5′-trihydroxyphenyl)-gamma-valerolactone and 5-(3′,4′-dihydroxyphenyl)-gamma-valerolactone. These metabolites have also been also monitored in other studies. Figure 1 demonstrates the chemical structure of the main flavanols (catechin and epicatechin) in cocoa and their metabolites.

**TABLE 2:** The concentration of flavanols in selected plant foods (Fernandez-Murga et al., 2011).

Plant food	Concentration of flavanols
Cocoa beans	1.4 g/kg
Chocolate	0.46–0.61 g/kg
Grape seeds	1.7 g/kg
Grapes	0.01 g/kg
Apples	0.03–0.1 g/kg
Apricots	0.01 g/kg
Green tea (200 mL)	100–800 mg/L
Black tea (200 mL)	60–500 mg/L
Red wine (100 mL)	80–300 mg/L

However, the bioavailability of flavanols is low, with a maximum plasma concentration rarely exceeding 1 µM (Andjuar et al., 2012). In addition, the degree of absorption varies between flavanols. For example, the concentration of the catechin monomer is less than 10% of that of epicatechin. However, different interactions with foods may change bioavailability and biological activity. For example, milk chocolate demonstrates lower antioxidant capacity than dark chocolate, which has been attributed to the presence of milk proteins. In addition, if a meal is rich in carbohydrates, absorption has been found to increase by up to 40%; however, no such change is observed if the mean is rich in proteins or lipids (Serafini et al., 2003; Fernandez-Murga et al., 2011; Loffredo et al., 2017). The bioavailability of cocoa phenolic compounds has been reviewed by Andjuar et al. (2012).

## Effect of cocoa and its products on blood platelet concentration

Although studies indicate that consumption of cocoa products may affect blood platelet count, their observations are sometimes controversial. For example, Calderon-Garciduenas et al. (2013) noted that consumption of dark chocolate (30 g with 680 mg of total flavanols, for 9–24 days) increases the number of blood platelets isolated from children who are exposed to air pollution. However, this increase was not statistically significant. Esser et al. (2014) report that consumption of 70 g normal and high flavanol chocolate for 1 month in obese men has no effect on blood platelet number. Another study revealed that four-week consumption of 50 g dark chocolate with low and high flavanols results in no change in blood platelet count in patients with chronic heart failure. Recently, Raguzzini et al. (2019) found no differences in blood platelet count between chocolate consumers (1–3 times/week) and non-consumers; however, the authors did not describe the kind of chocolate or its chemical content.

## Cardioprotective mechanisms of cocoa phenolic compounds

Many studies indicate that cocoa phenolic compounds are bioactive and demonstrate cardioprotective properties deriving from several mechanisms. The cardioprotective properties of phenolic compounds, especially flavanols and other flavonoids, are partially attributed to their antioxidant and anti-inflammatory properties (Anger et al., 2016). Cocoa phenolic compounds have been also confirmed to have anti-platelet properties in a number of studies (Holt et al., 2006; Almoosawi et al., 2012; Andjuar et al., 2012; Anger et al., 2016; Vlachojannis et al., 2016; Dong et al., 2017; Lee et al., 2017; Loffredo et al., 2017; Latif and Majeed, 2020; Seecheran et al., 2022). For example, Kim et al. (2017) report that cocoa phenolic compounds exert potent anti-platelet properties both directly and indirectly via endothelial cells, and conclude that they have the potential for lowering the risk of CVD-related hypercoagulation due to hypercholesterolemia.

Murphy et al. (2003) observed that after 28-day exposure to flavanols and procyanidins from cocoa resulted in an increase of catechin and epicatechin concentration in plasma (by 28% and 81%, respectively), and a decrease of blood platelet function.

Another recent paper by D’Amico et al. (2022) studied the relationship between blood platelet activation, oxidative stress and muscular injuries stimulated by physical exercise, as well as the role of cocoa-derived phenolic compounds, in elite athletes. Their results suggest that the cocoa-derived phenolic compounds: catechin and epicatechin significantly reduce oxidative stress and muscle injury in supernatants of human skeletal muscle myoblast cell cultures treated with plasma. They observed downregulation of NADPH oxidase 2 (NOX2) activation, H_2_O_2_ production and reduction of creatine kinase (CK) and α-actin after cell treatment. However, the authors do not indicate the concentrations of the phenolic compounds.

A recent study by Li R. et al. (2022) found that flavonoids exert anti-inflammatory properties in ischemic stroke by acting as modulators of microglia polarization *via* the toll-like receptor (TLR4)/nuclear factor-κB (NF-κB) signaling pathway. Catechin, an important cacao flavonoid, facilitates the cardioprotective role of nuclear factor-erythroid 2-related factor 2 (Nrf2) and its downstream molecules through *inter alia* NF-κB, glutathione peroxidase and kinases (Talebi et al., 2021). Moreover, quercetin, another phenolic compound, can demonstrate cardioprotective potential by affecting molecular pathways such as phosphoinositide 3–kinase (PI3K)/protein kinase B (Akt) and mitogen-activated protein kinase (MAPK) (Askrafizadeh et al., 2021; Li Q. et al., 2022).

**FIGURE 2:**
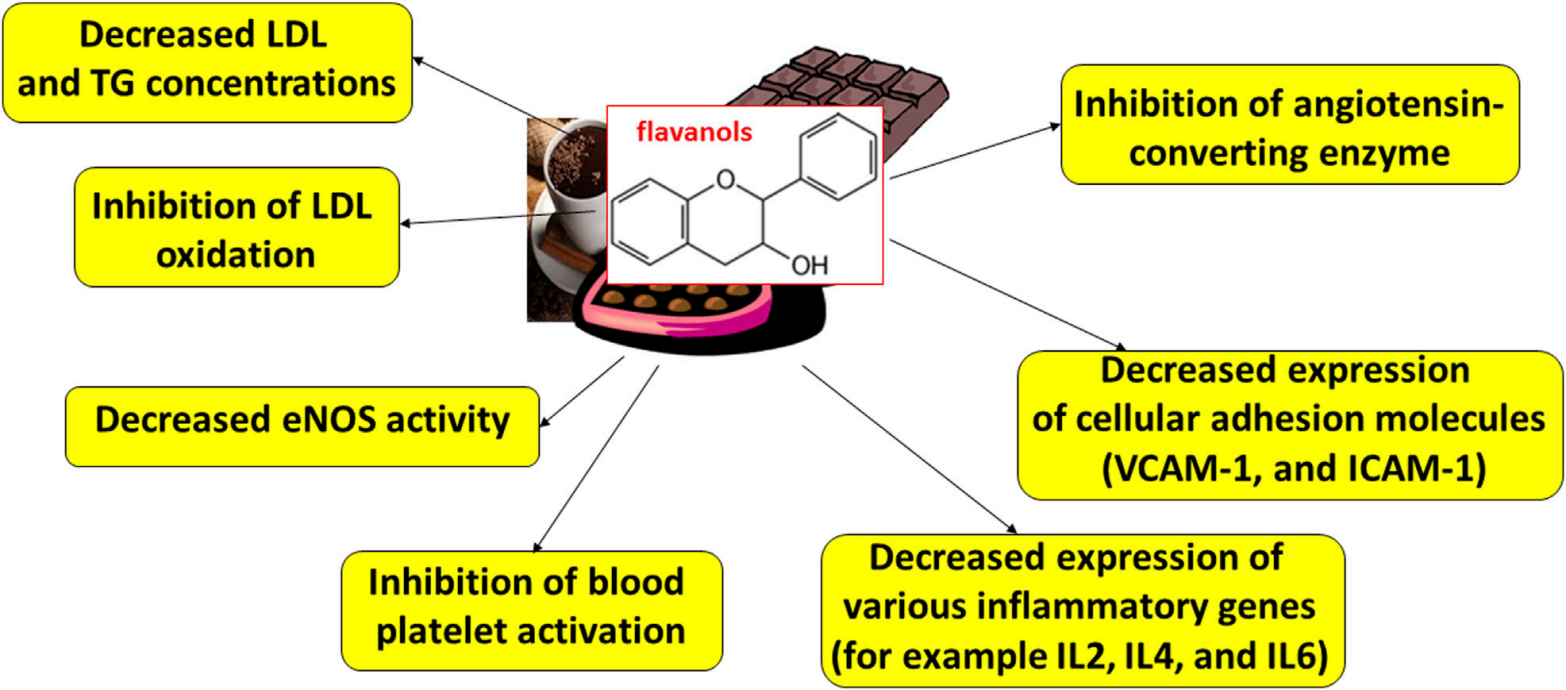
Cardioprotective mechanisms of flavanol-rich dark chocolate (Rees et al., 2018). eNOS, endothelial nitric oxide synthase; ICAM-1, intracellular adhesion molecule 1; IL, interleukin; LDL, low-density lipoprotein; TG, triglycerides; VCAM-1, vascular cell adhesion molecule 1. More details in text.

Proposed cardioprotective mechanism of action of flavanols from cocoa and its products is presented on Figure 2. For example, these compounds may induce a decrease of reactive oxygen species and nitric oxide production. The decrease of intracellular ROS level may be accompanied by the recovery of phosphatase activity. Reactivated phosphatases may inhibit the activity of tyrosine kinases, and thus blood platelet aggregation (by inhibition of GPIIb/IIIa exposition). These compounds may also reduce the activity of cyclooxygenase and reduce arachidonic acid metabolism. Moreover, consumption of flavonol-rich dark chocolate is believed to result in decreased low-density lipoprotein (LDL) and triglyceride (TG) concentrations, inhibition of LDL oxidation, reduced endothelial nitric oxide synthase (eNOS) activity, and reduced expression of several inflammatory genes (interleukins and TNF-α); it also reduces the expression of cellular adhesion molecules such as intracellular adhesion molecule 1 (ICAM-1) and vascular cell adhesion molecule 1 (VCAM-1), and inhibition of angiotensin-converting enzyme (Almoosawi et al., 2012; Andjuar et al., 2012; Anger et al., 2016; Vlachojannis et al., 2016; Dong et al., 2017; Kim et al., 2017; Lee et al., 2017; Loffredo et al., 2017; Latif and Majeed, 2020; Seecheran et al., 2022) (Figure 2). However, further studies are needed to clarity the cardioprotective mechanisms of their action.

## Anti-platelet properties of cocoa phenolic compounds and cocoa products

Blood platelet functions may be determined by various approaches based on blood platelet aggregation, blood platelet adhesion, ATP release or exposure of the activated conformation of glycoprotein (GP) IIb/IIIa and P selectin. A number of methods can be used to achieve this including blood platelet analysis and flow cytometry based on a range of biological materials, particularly whole blood, platelet-rich plasma, and washed blood platelets (Sang et al., 2021).

About 20 years ago, Rein et al. (2000) observed that consumption of cocoa, containing 897 mg total epicatechin and olimeric procyanidin, decreases platelet aggregation stimulated by various agonists, *viz.* ADP, collagen, and adrenaline. A similar effect was observed after consumption of moderate amounts of cocoa phenolic compound (220 mg). These anti-aggregatory properties were associated with reduced exposure of the activated conformation of GPIIb/IIIa on the blood platelet surface, revealed by a PFA100 platelet function analyzer (*ex vivo* model). Similarly, Pearson et al. (2002) found that flavanol-rich cocoa has an inhibitory effect on the activation of blood platelets stimulated by epinephrine (*ex vivo* model).

Flammer et al. (2007) found blood platelet adhesion to be reduced 2 hours after consumption of dark chocolate containing 0.27 mg/g of catechin an 0.9 mg/g of epicatechin, and a total phenolic compound content of 15.6 mg of epicatechin equivalent per Gram. This effect was observed in 22 heart transplant recipients in a double-blind, randomized study (*ex vivo* model).

Carnevale et al. (2012) found that dark chocolate reduces blood platelet activation by lowering oxidative stress in smokers; however, no such effect was observed in healthy subjects. Twenty healthy subjects and smokers received 40 g of dark chocolate (cocoa >85%) or milk chocolate (cocoa <35%). The level of oxidative stress was measured by different biomarkers, including generation of reactive oxygen species (ROS) and eicosanoids (*in vivo* model).

A recent meta-analysis of randomized clinical trials by Azad et al. (2021) found consumption of cocoa products to have beneficial effects on blood platelet functions in healthy adults regardless of age; this was especially true in men and when consumption was for 4 weeks or longer. This review paper included 21 articles, and 388 participants, including both non-smokers and smokers, as well as individuals with mild hypertension, chronic heart failure, and postmenopausal hypercholesterolemia. Moreover, a randomized clinical trial by Sesso et al. (2022) found that while cocoa supplementation has no effect on the primary outcome of total CV events, consumption significantly reduces CV mortality by 27%.

A recent paper by Seecheran et al. (2022) examined the effect of consumption of dark chocolate (30 g/day; 65% cocoa, for 1 week) on blood platelet function in patients with coronary artery disease (*n* = 20) receiving maintenance dual anti-platelet therapy: clopidogrel (75 mg/day) and aspirin (81 mg/day). Blood platelet function was determined by aspirin reaction unit (ARU) assays and the VerifyNow P2Y2 reaction unit (PRU) using blood sample. The authors indicate that consumption of dark chocolate augments the inhibitory effect of clopidogrel, but not aspirin (*in vivo* model).

**FIGURE 3:**
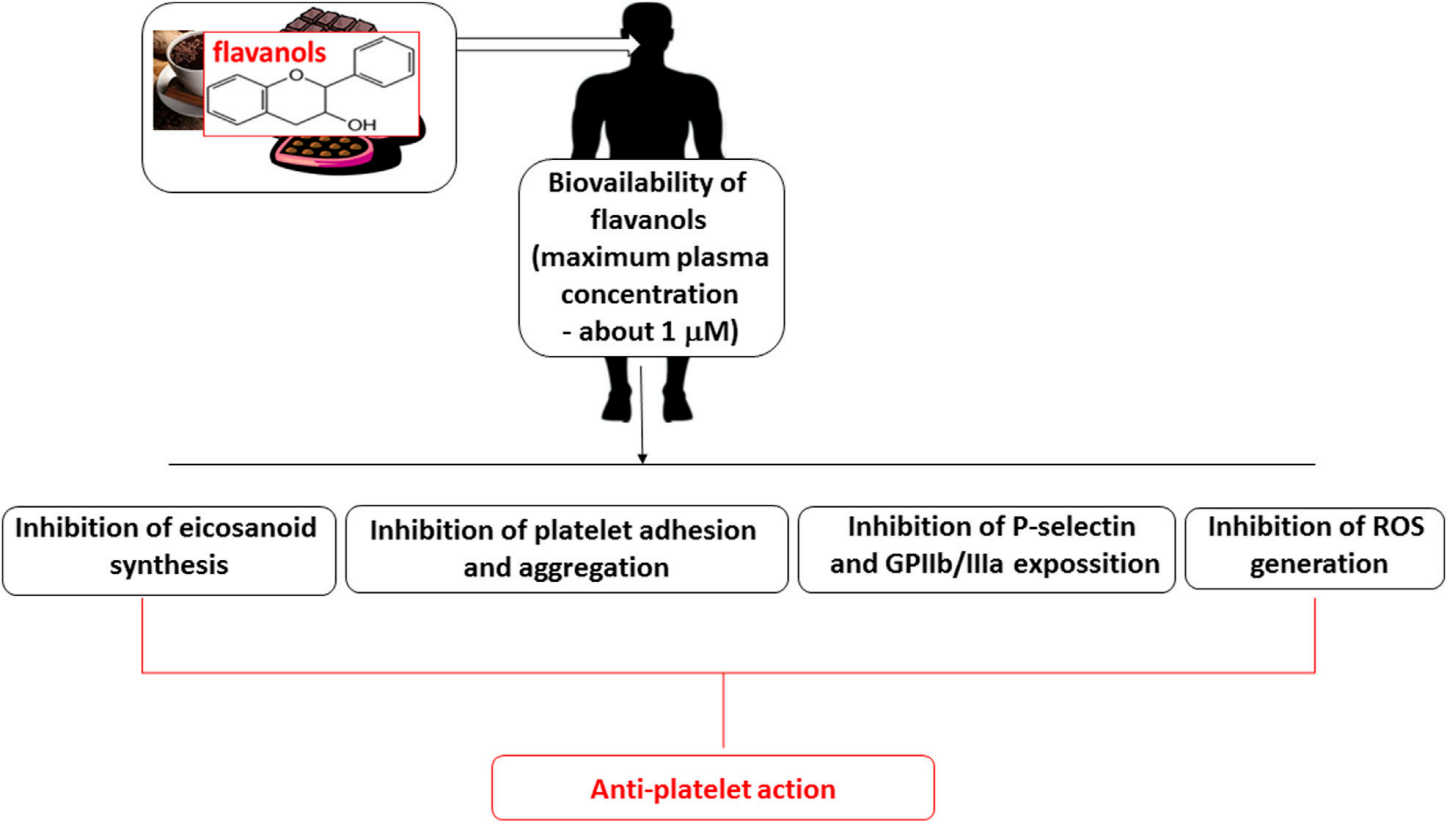
Proposed anti-platelet action of cocoa flavanols.

The anti-platelet potential of cocoa and its various food products, as indicated in other *ex vivo* and *in vivo* models, are summarized in Table 3 and Figure 3. It is an important to note that many of the studies presented in Table 3 were based on flavanol-rich cocoa administration in both healthy subjects and patients with mild hypertension or coronary artery problems. However, their anti-platelet activity appears to be dependent on various factors, including the type of cocoa product and its chemical content, the method used for measuring blood platelet function, and the type of blood platelet agonist. Moreover, many studies fail to describe the glucose, protein, lipid and caloric content of the tested chocolate, or to demonstrate the plasma concentrations of flavanols with relatively low bioavailability; in addition, their downstream plasma metabolite concentrations may not correlate with their positive effects on CVDs. Many studies fail to consider the effects of long-term consumption. Therefore, there is a need for longer studies examining the anti-platelet action and safety of cocoa flavanols and other components.

**TABLE 3:** Anti-platelet properties of cocoa and its products in various *ex vivo* and *in vivo* models.

Cocoa and its products	Size group	Time	Subjects	Biological material/method for platelet activation/Parameters of platelet activation	References
*Ex vivo*					
Cocoa (containing 220 or 897 mg total epicatechin and oligomeric pracyanidin)	30	—	Healthy subjects (blood was obtained before 2 and 6 after ingestion of cocoa)	Blood/Flow cytometry/A platelet function analyzer/Platelet aggregation stimulated by ADP, collagen or adrenaline (inhibition)	Rein et al. (2000)
				GPIIb/IIIa exposure stimulated by these agonists (inhibition)	
Dark chocolate (70% cocoa), 40 g	22	—	Subjects after heart transplant (blood was obtained before 2 h after ingestion of cocoa)	Blood/A platelet function analyzer/Blood platelet adhesion (inhibition)	Flammer et al. (2007)
Flavanol-rich cocoa beverage (18.75 g of flavanol-rich cocoa powder), 300 mL	16	—	Healthy subjects	Blood/An analyzer (the PFA-100)/GPIIb/IIIa exposure stimulated by ADP (inhibition)	Pearson et al. (2002)
Flavan-3-ol-enriched dark chocolate/60 g	42	—	Healthy subjects	Blood/A platelet function analyzer/Platelet aggregation stimulated by ADP (inhibition)	Ostertag et al. (2013)
***In vivo***					
Tablet (39 mg cocoa flavanols); 6 tablets/day	13	28 days	Healthy (non-smokers)	Blood/Flow cytometry/Platelet aggregation stimulated by ADP or collagen (inhibition)	Murphy et al. (2003)
				P selectin exposure stimulated by ADP (inhibition)	
				ATP release induced by ADP or collagen (inhibition)	
Dark chocolate (85% cocoa) 40 g/day	20	20 days	Healthy subjects (smokers)	Platelet-rich plasma (PRP)/ROS and eicosanoid generation (inhibition)	Carnevale et al. (2012)
Dark chocolate, 16 g/day	23	28 days	Healthy subjects	Urine/Arachidonic acid metabolism (no effect)	Wan et al. (2001)
Milk chocolate, 96 g/day	9	21 days	Healthy subjects	Blood/A platelet function analyzer/Platelet aggregation stimulated by ADP (inhibition)	Kelly et al. (2002)
Dark chocolate (75% cocoa), 100 g/day	9	14 days	Healthy subjects	Platelet-rich plasma (PRP)/Platelet aggregation stimulated by collagen (inhibition)	Innes et al. (2003)
Cocoa (240 mg/day)	8	7 days	Healthy athlete	Blood/Flow cytometry/P selectin exposure stimulated by ADP (inhibition)	Singh et al. (2006)
High-cocoa flavanol (750 mg/day)	16	28 days	Coronary artery disease patients	Blood/Flow cytometry/Platelet microparticles formation (no effect)	Horn et al. (2014)
High flavanol dark chocolate (1,064 mg flavanols), 50 g/day	26	42 days	Subjects with mild hypertension	Platelet-rich plasma (PRP)/Platelet aggregation stimulated by ADP and thrombin receptor activating peptide; (TRAP) (inhibition)	Rull et al. (2015)

A review by Holt et al. (2006) indicates that flavanol-rich food products, including cocoa and its products, may modulate various elements of hemostasis; for example, the authors suggest that these compounds can inhibit blood platelet adhesion and aggregation, ROS generation, eicosanoid synthesis, P-selectin and GPIIb/IIIa exposure.

The proposed mechanism of action of cocoa flavanols on blood platelets is presented in Figure 3. The mechanism by which the phenolic compounds in cocoa, especially flavanols, inhibit blood platelet function remains complex and ambiguous. It is possible that flavanols decrease ROS generation and inhibit eicosanoid biosynthesis and platelet aggregation by inhibiting GPIIb/IIIa exposure. However, without knowing at least one direct cellular target of flavanols and their metabolites, their specific mode of action remains unclear. Therefore, further studies are needed to clarify the mechanism of their anti-platelet action.

## Conclusion

In recent years, cocoa and its products have been objects of various studies examining their cardioprotective action. Their findings demonstrate that cocoa consumption, especially in the form of dark chocolate, with a high flavanol concentration, has anti-platelet activity and may play a significant role in cardioprotection; they also note that cocoa consumption may be a good strategy in diminishing cardiovascular risk, including hyperactivation of blood platelets (Fernandez-Murga et al., 2011; Andjuar et al., 2012; Senturk and Gunay, 2015; Loffredo et al., 2017; Zięba et al., 2019).

Studies have described the interactions between chocolate and various drugs, such as antibiotics and statins (Antal et al., 2001; Piotrowicz et al., 2008; Scolaro et al., 2018), and these interactions are considered crucial for the efficacy of treatment in different diseases. In the case of the two antiplatelet drugs aspirin and clopidogrel, Collyer et al. (2009) suggest that chocolate acts synergistically with them by the inhibition of cyclooxygenase–I (COX-I), and this may lead to bleeding. Recently, Seecheran et al. (2022) found that the consumption of dark chocolate augments the inhibitory effect of clopidogrel in patients with coronary artery disease. However, little is known of the precise mechanisms behind the interactions of antiplatelet drugs or supplements with cocoa, its products (especially chocolate) and components (e.g., phenolic compounds and lipids), or the absorption of anti-platelet drugs and supplements after consuming cocoa and its products. Hence, the effect of cocoa on platelet activity, and the question of whether it may be part of a sweet strategy in diminishing CVDs, remains open.

## Abbreviations

ARU, aspirin reaction unit; CK, creatine kinase; COX-1, cyclooxygenase-1; CVDs, cardiovascular diseases; eNOS, endothelial nitric oxide synthase; GAE, gallic acid equivalent; GP, glycoprotein; ICAM-1, intracellular adhesion molecule 1; IL, interleukin; LDL, low-density lipoprotein; NOX2, NADPH oxidase 2; PFA100, platelet function analyzer; PRU, P2Y2 reaction unit; ROS, reactive oxygen species; TG, triglycerides; TRAP, thrombin receptor activating peptide; VCAM-1, vascular cell adhesion molecule 1.
